# Supplementary material for: Yes-Associated Protein (Yap) Is Up-Regulated in Heart Failure and Promotes Cardiac Fibroblast Proliferation
Source: Int J Mol Sci. 2021 Jun 7;22(11):6164. doi: 10.3390/ijms22116164 (PMC8201133; doi:10.3390/ijms22116164)
Supplement: Supplementary file 1 [file ijms-22-06164-s001.zip › ijms-1089600-supplementary.pdf]

Supplemental Figures

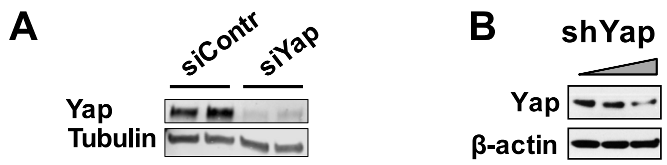

Supplemental Figure S1. Efficient Yap downregulation. (**A**) Human adult cardiac fibroblasts were transfected with control (siContr) and Yap (siYap) siRNA, followed by immunoblot analysis to Yap protein level; $n = 5$. (**B**) Human adult cardiac fibroblasts were transduced with different doses of adenovirus expressing shRNA Yap (shYap) followed by immunoblot analysis to detect Yap; $n = 2$.

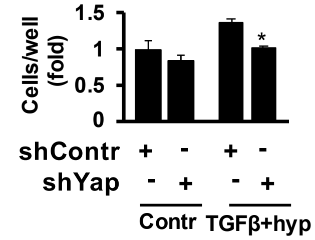

Supplemental Figure S2. Yap contributes to human adult cardiac fibroblast growth. Cardiac fibroblasts infected with adenovirus expressing shRNA Yap (shYap) or control adenovirus (shContr) and exposed to hypoxia (1% O2) and TGFβ (10 ng/mL) followed by cell count performed at day 5 of exposure. Data are mean ± S.E.M; * and # $p < 0.05$ vs. shContrl and shContrl+hypoxia/TGFβ, respectively, n = 3–4/condition.

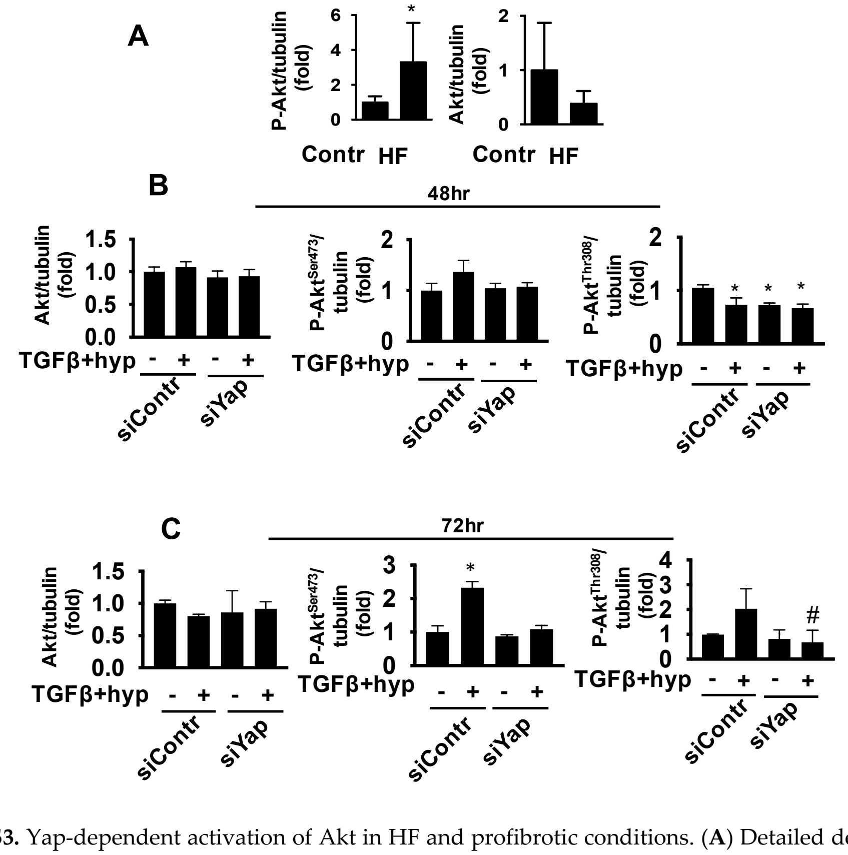

Supplemental Figure S3. Yap-dependent activation of Akt in HF and profibrotic conditions. (**A**) Detailed densitometry analysis of LV tissues from HF patients and control individuals from Figure 4A showing quantification of Akt and P-Akt normalized to

housekeeping control. Data represent mean ± S.E.M of fold changes from control; $n = 7$ subjects/group, $*p < 0.05$ vs. control (**B**,**C**) Graphs representing fold change quantitative densitometry analysis normalized to corresponding loading control for total and phospho-Akt from Figures 4C–F of human cardiac fibroblasts transfected with siContr or siYap exposed to normoxia and vehicle (-) or hypoxia (1% O_2) and TGFβ (10 ng/mL) for stated durations. Data represent mean ± S.E.M. of fold changes from siContr control (-); $*p < 0.05$ vs. siContr control (-),$\#p < 0.05$ vs. siContr control exposed to hypoxia (1% O_2) and TGFβ (10 ng/mL); $n = 3$–4/condition.

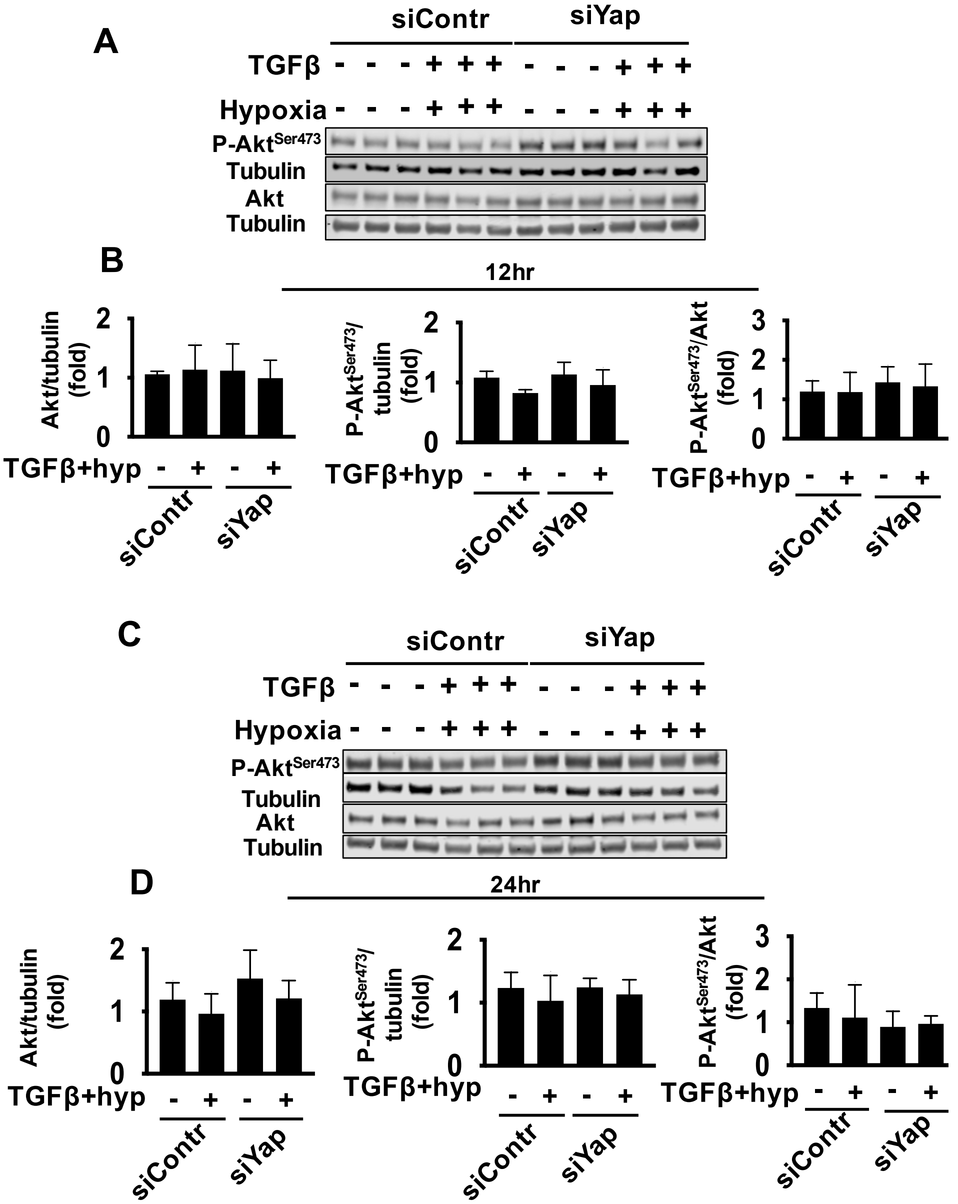

Supplemental Figure S4. Akt is not activated at early profibrotic stress timepoints. (**A**,**C**) Representative immunoblot for indicated proteins of human adult cardiac fibroblasts transfected with siYap or siContr and exposed to hypoxia/TGFβ. (**B**,**D**) Fold change immunoblot densitometry analyses of indicated proteins normalized to corresponding loading control. Data represent mean ± S.E.M of fold changes to siContr-infected vehicle-treated control group (-); $n = 3$/condition.

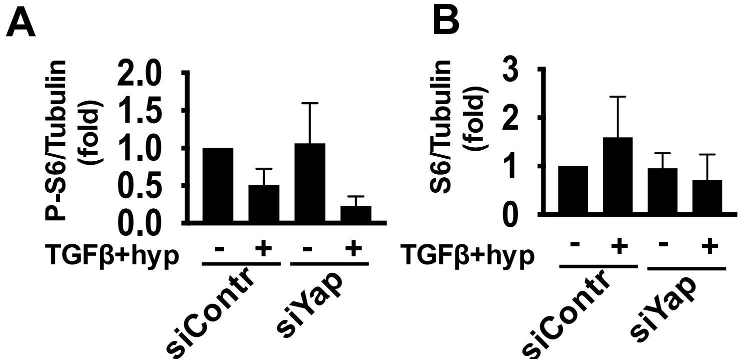

Supplemental Figure S5. Alleviation of mTOR signaling with profibrotic conditions does not alter with Yap silencing. (**A**,**B**) Fold change immunoblot densitometry analyses from Figure 5A of S6 and phospho-S6 (*p*-S6) normalized to corresponding loading control in human adult cardiac fibroblasts transfected with siYap or siContr and exposed to hypoxia/TGFβ. Data represent mean ± S.E.M of fold changes to siContr-infected vehicle-treated control group (-); $n = 3–4$/condition.

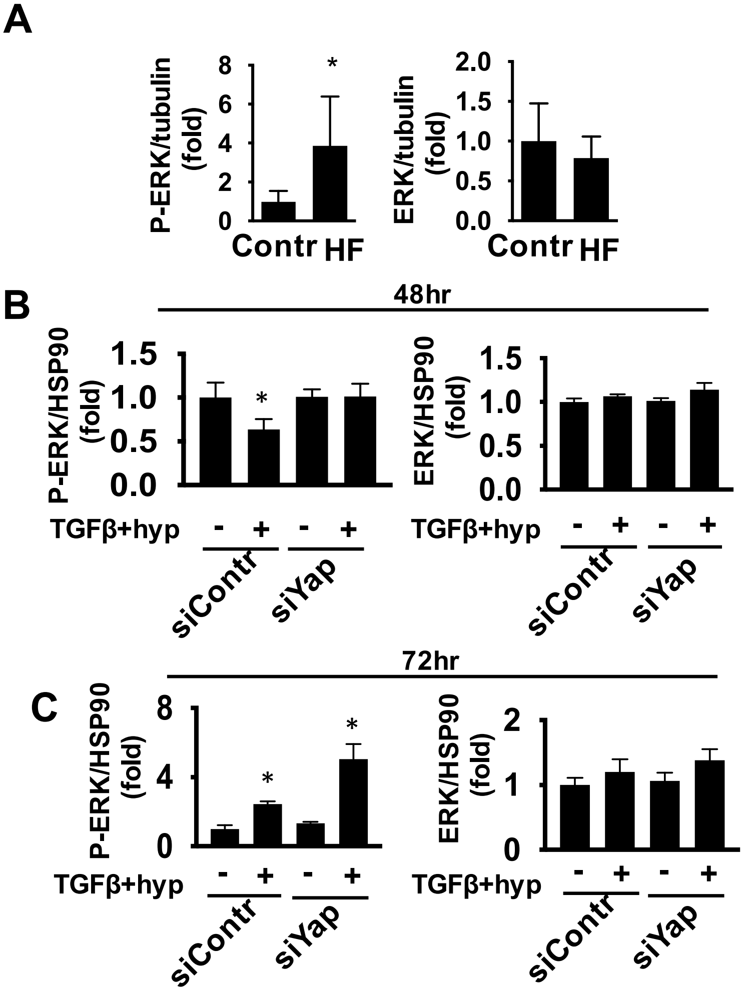

Supplemental Figure S6. Yap-independent elevation of ERK1/2 signaling under conditions of HF. (**A**–**C**) Quantitative densitometry analyses of indicated proteins in (A) LV tissues from HF patients and control individuals from Figure 6 A. Data represent mean ± S.E.M of fold changes from control; $n = 7$ subjects/group, $^*p < 0.05$ vs. control. (**B**,**C**) Human adult cardiac fibroblasts transfected with siYap or siContr and exposed to hypoxia/TGFβ for stated durations normalized to corresponding loading control from Figures 6C,E. Data represent mean ± S.E.M of fold changes from siContr-infected vehicle-treated control group (-); $n = 3–4$/condition, $^*p < 0.05$ vs. corresponding (-) control.

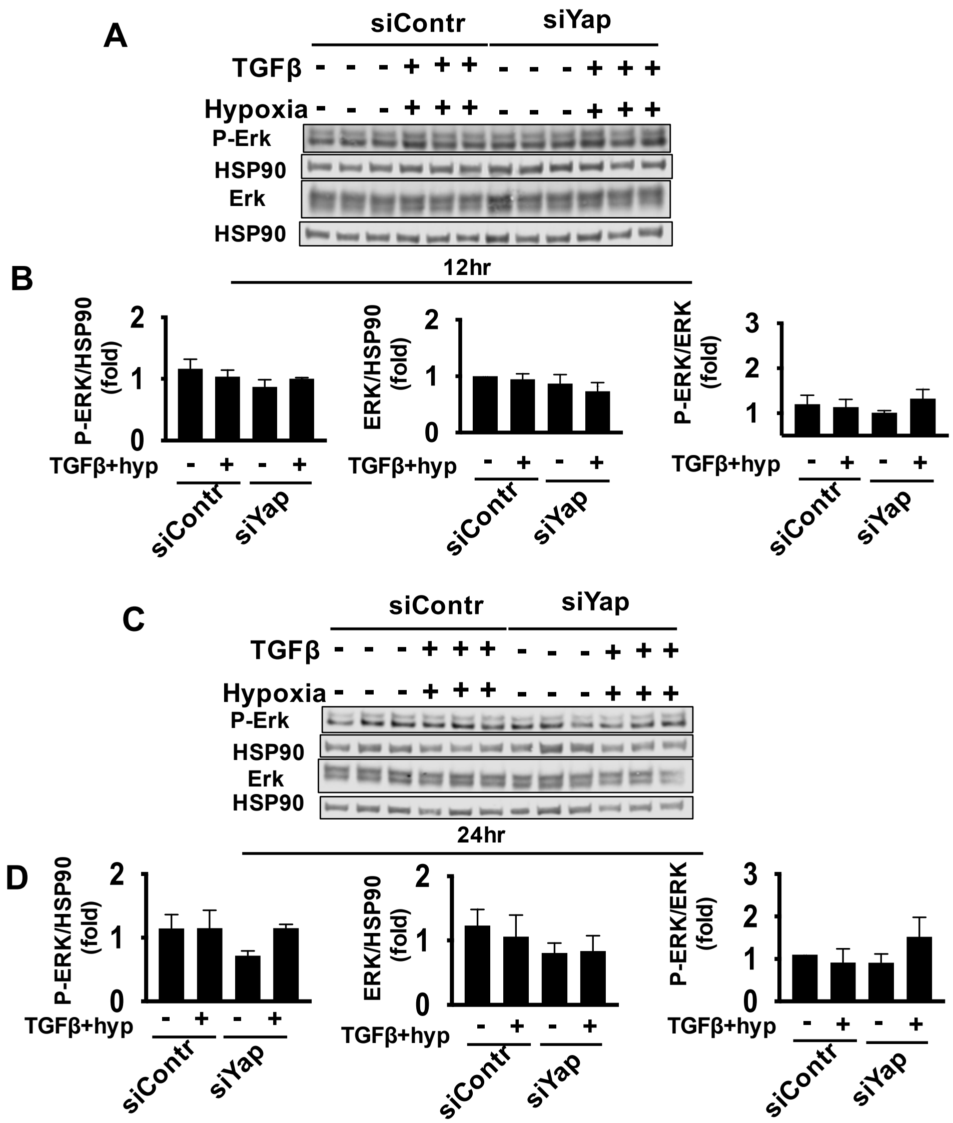

Supplemental Figure S7. Hypoxia/TGFβ does not activate Erk signaling at early timepoints. (**A**,**C**) Representative immunoblot for indicated proteins of human adult cardiac fibroblasts transfected with siYap or siContr and exposed to hypoxia/TGFβ. (**B**,**D**) Fold change immunoblot densitometry analyses of indicated proteins normalized to corresponding loading control. Data represent mean ± S.E.M of fold changes to siContr-infected vehicle-treated control group (-); *n* = 3/condition.
